# Effect of Action Video Games in Eye Movement Behavior: A Systematic Review

**DOI:** 10.16910/jemr.17.3.6

**Published:** 2024-09-25

**Authors:** Anna Montolio-Vila, Marc Argilés, Bernat Sunyer-Grau, Lluïsa Quevedo, Graham Erickson

**Affiliations:** Universitat Politècnica de Catalunya, Terrassa, Spain; Pacific University, Oregon, United States of America

**Keywords:** Eye movements, action video games, systematic review, eye tracking, fixations, saccades, smooth pursuit, attention

## Abstract

Previous research shows that playing action video games seems to modify the behavior of eye movements such as eye fixations and saccades. The aim of the current work was to determine the effect of playing action video games on eye movements behavior such as fixations, saccades and pursuits. A systematic research review in PubMed and Scopus databases was conducted to identify articles published between 2010 and 2022 which referred to action video games and eye movements, including fixations, saccades and pursuits. We included those that were experimental and quasi-experimental, comparing at least two groups between action vs. non-action video games players. All the studies included used an eye tracker to study eye movements. A total of 97 scientific articles were found in the databases. After inclusion criteria, thirteen articles (N=13) were analyzed for the present work, of which ten (n=10) had a cross-sectional design, and three (n=3) were randomized intervention studies. Playing regularly or training with action video games is not likely to produce changes in eye movements, based on the literature research analyzed. For future research, more interventional studies, with less gender bias, more sample participants and general consensus on the distinction between the action and non-action video games is needed.

## Introduction

According to a report by DFC Intelligence, almost 40% of the world's population plays video games, which represents about 3.1 billion people participating in the gamer scene (19). Of all video game genres and styles, action video games (AVG) are the most studied by the scientific community. Research interest in AVG likely arises from the specific characteristics these games possess, as in the meta-analysis of Bediou et al. (9) stated: (a) a fast pace (in terms of the speed of moving objects, the presence of many highly transient events, and the need to execute motor responses within severe time constraints); (b) a high degree of perceptual and motor load, and cognitive requirements such as working memory, planning, and goal setting (e.g., multiple items to track simultaneously, different possible goals that must be constantly reassessed, and countless motor plans that must be executed quickly); (c) a constant need to switch between a highly focused state of attention (e.g., toward directed goals) and a more distributed state of attention (e.g., to monitor the entire field of vision); and (d) a high degree of clutter and distraction (i.e., items of interest are distributed among many non-target items). Moreover, players' abilities in domains such as hand-eye coordination and reaction time are key for performance in these types of video games.

Within AVG are found a wide variety of subgenres, including fighting and shooting games. Multiplayer online battleground arena (MOBA) and some real-time strategy games are also considered action games by some authors (6). Studies have investigated the impact of AVG on various cognitive and perceptual domains in an effort to identify which skills can be more reliably modified (12; 28). In a meta-analysis, Bediou et al. (9) confirmed that habitually playing AVG had a medium impact size on cognition, and that training inexperienced young adults in AVG had a small to medium effect in some cognitive domains. AVG are an attractive tool for investigating the limits of neuroplasticity changes in perception, attention, and cognition, opening new insights on methods to foster learning and brain plasticity in a wide variety of tasks and domains (28).

Studies have also used AVG for cognitive rehabilitation in patients with traumatic brain injuries with positive results (62). Green & Bavelier (26) demonstrated how training with AVG was able to modify some visual functions such as visual attention (26). Subsequently, other studies have demonstrated improvements in visual acuity, stereopsis, spatial attention and contrast sensitivity function in subjects with amblyopia (41; 40). Improvements have also been studied in people with dyslexia, in whom AVG training appears to improve processing speed and reading (21; 23). A systematic review of the literature showed that AVG are a promising treatment for dyslexia, leading to gains in reading pace and fluency (53). Moreover, developing specific visual skills through games is particularly attractive, since it promotes motivation and engagement, and may facilitate learning processes (3).

In video games, eye movements have been studied with eye trackers for two main objectives. First, as a way of navigating through the video game, replacing the traditional methods (joystick, mouse, keyboard), and making games more accessible to people with motor difficulties. And second, as a method of analysis and evaluation of video game expertise (1; 57; 59). Eye movements can be studied with a variety of metrics. For fixations, metrics include the number of fixations in a specific space and time, the distribution of fixations in a specific space, and fixation duration (in milliseconds). For saccadic eye movements, metrics include speed, latency, gain, number and amplitude. For pursuit eye movements, metrics include speed and precision.

Several studies have highlighted a notable correlation between regular engagement in action video games (AVG) and improvements in eye movement behaviors, specifically in metrics such as saccadic latency and gain (12; 31). This observation is particularly interesting, in light of the established connection between eye movements and cognitive processes (30; 35; 38; 42; 46; 47; 49) and ito study reading performance (11; 25; 54; 60; 64). Moreover, a body of research consistently suggests that playing AVG enhances visual attention abilities (7; 9; 26; 62) and reading performance (21; 23).

The fundamental objective is to explore the potential relationship between consistent playing of AVG and the possible modifications in eye movement behavior. By synthesizing existing evidence and delving into the intricacies of eye movement dynamics, this investigation aims to contribute to the understanding of how either regularly playing or training with AVG can change eye movement metrics in terms of fixations, saccades, and pursuits, and to provide nuanced insights into the impact of AVG playing on the visual and cognitive aspects of human behavior.

The goal of this systematic review was to investigate studies that measured the possible modifications seen in eye movements (fixations, saccades and pursuits) through regular experience or treatment with AVG.

## Methods

Eye movement metrics in eye fixations can be analyzed with duration, which consists of the time of each or grouped fixation, and number and distribution depending on the region of interest (ROI) during a specific task (18; 50). Saccadic movements are characterized by gain, defined as a saccade amplitude divided from target amplitude; latency, the difference between the appearance of stimuli and execution of saccadic movement; speed or velocity, measured in milliseconds, the number of saccades during a specific task or ROI; and amplitude, the distance traveled by a saccade. The main sequence in saccadic is defined by the relationship between saccadic amplitude and peak velocity (51), and usually has a linear relationship (33). Pursuit movements can be defined by amplitude or length, speed or velocity, and smoothness during a task.

Further, this review focused on identifying studies whose objectives were:

1. To observe the possible differences in eye movements between experienced players in AVG compared with non-action video game (NAVG).

2. To observe changes in eye movements metrics and behavior before and after cognitive treatment using AVG.

### Design and Procedure

Search results were restricted to articles published between 2010 and 2022. This inclusion was determined for a better homogeneity in video games and eye tracking technology used in research. All publications referring to AVG regardless of the specific type of action video game were selected for further examination. For inclusion in the review, we concentrated on experimental and quasi-experimental studies, those comparing AVG vs. NAVG, those using eye tracker devices to measure the quality of eye movements, and those with participants without specific pathologies. The outcomes data sought were: a) fixation duration, number and distribution, b) saccadic eye movement amplitude, velocity, latency, and c) pursuit eye movement velocity, latency and precision. Two of the authors (A.M.V and M.A) conducted the study selection and data collection during July to November 2022. This research was carried out using PubMed and Scopus databases, using the following Boolean search terms:

● eye movements AND eSports

● eye movements AND action videogames

● action AND video AND game AND fixation

● action AND video AND game AND pursuits

● action AND videogame AND saccades

● saccadic AND movements AND videogames

● saccades AND videogames

**Figure 1.**
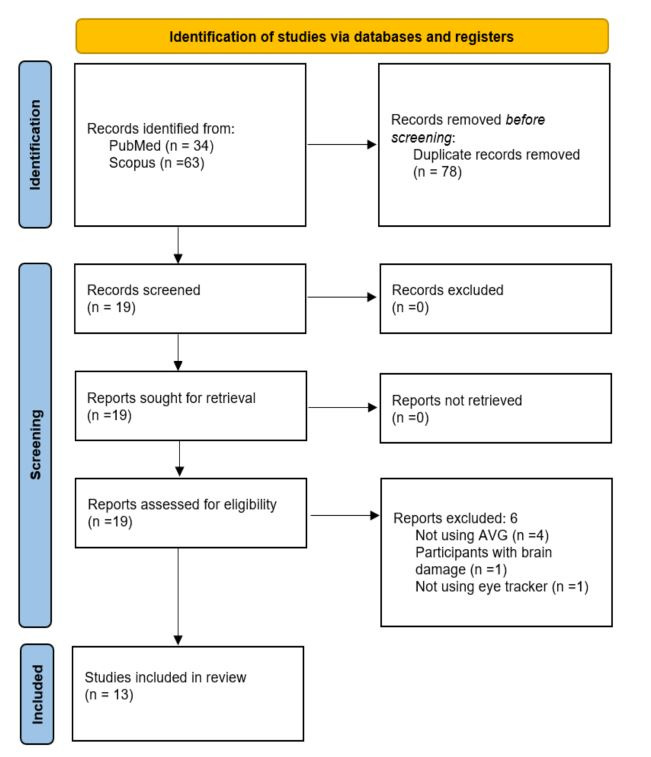
PRISMA flow diagram of the screening process.

This systematic review was registered in the international register of systematic reviews PROSPERO with registration number CRD42022358557. Flow chart diagram of the screening process is shown in Figure 1.

The tool used to assess the risk of bias in the randomized studies was ROB-2, which provides a framework for considering the risk of bias in the findings of any type of randomized trial. This tool is structured in five domains through which bias can be introduced into the result. These were identified based on both empirical evidence and theoretical considerations [19]. Each domain contains a series of questions, "flag questions", aimed to elicit information about trial characteristics that are relevant to the risk of bias. This review was conducted in line with the Preferred Reporting Items for Systematic Reviews and Meta-Analyses (PRISMA) guidelines (Table 1 in supplemental material)

## Results

A total of 97 scientific articles were found in the databases (see Figure 1). Search results were as follows: eye movements AND eSports (5 results), eye movements AND action videogames (9 results), action AND video AND game AND fixation (47 results), action AND video AND game AND pursuits (24 results), action AND videogame AND saccades (2 results), saccadic AND movements AND videogames (4 results) and saccades AND videogames (6 results). Thirteen (N=13) of these articles met the inclusion criteria for the type of design, player comparisons and use of an eye tracker. Studies removed (6 results) were research which used multi-genre videogames, only studied participants playing general videogames, had participants only viewing videogames, used a driving simulator, studied patients with brain damage, or those not employing an eye tracker.

Detailed information was extracted from each of the thirteen incorporated studies, including: characteristics of the sample (number of participants, age and gender), study design, inclusion and exclusion criteria, video game and eye tracker used, and study results. This information is shown in Table 1. Ten (n=10) of the thirteen studies used a cross-sectional design, and three (n=3) were randomized intervention studies. In the following section we will summarize the results of the systematic review.

**Table 1.**
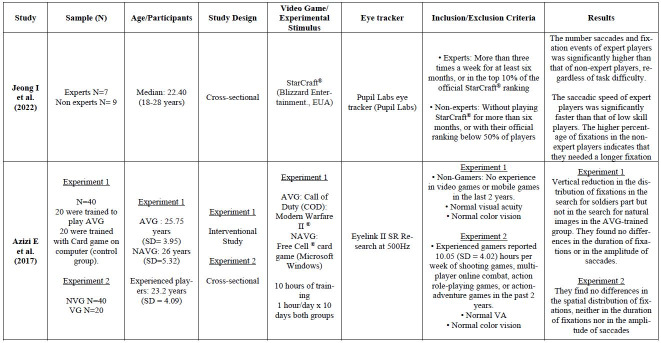

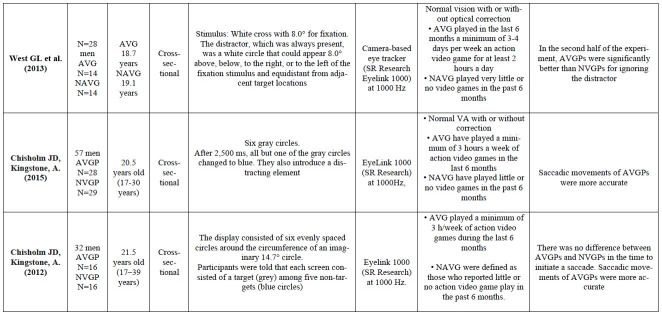

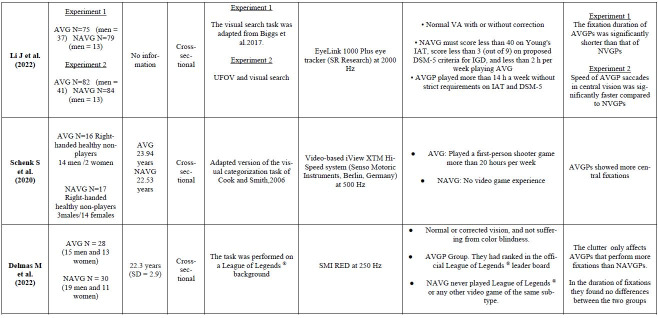

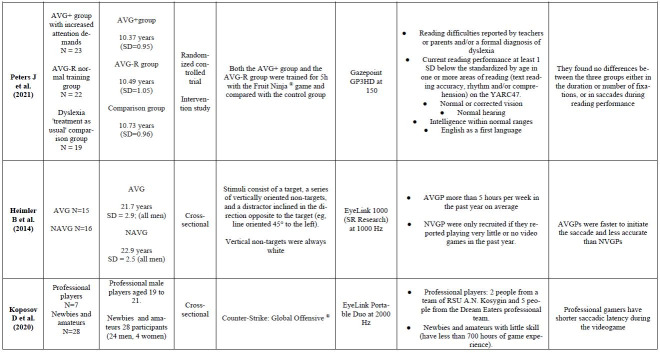

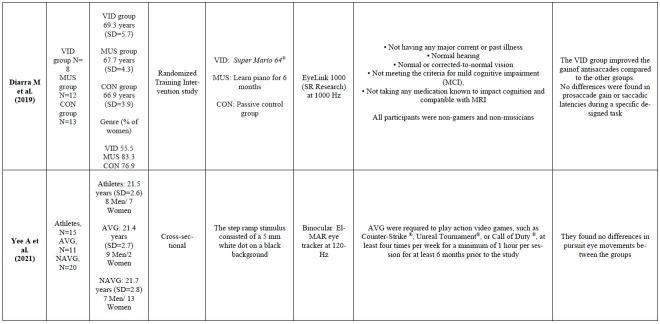
Detailed information of the studies included in the systematic review. .VA= Visual Acuity, AVG=Action Video Game, NAVG= Non-Action Video Game

### Eye fixations

#### Duration

Chisholm & Kingstone (12) compared 16 participants in action video game players (AVGP) and 16 in non-video game players (NVGP) in an oculomotor capture task. The authors asked subjects to make a saccadic eye movement towards the target, encouraging them to do it as quickly and as accurately as possible. They were not informed that distracting elements would appear that would make the task more difficult. They found no differences between groups. Delmas et al.,(17) studied how the presence of an excessive amount of information and the variability of this information in the visual scene affects visual search. In that experiment, the well-known game League of Legends^®^ was used as background. AVGP were defined as ranked in the official leader board of the videogame, and NVGP without experience in League of Legends^®^. No significant differences were found between groups in the duration of eye fixations during the videogame. Also, Azizi et al.(5) did not find differences in the duration of fixations in either of their two experiments. In their first experiment, they compared a cross-sectional study experience of players in a Call of Duty^®^ video game with NVGP. In their interventional study, 20 participants were trained with Call of Duty^®^ for 10 hours and 20 were trained with card games as a control group.

Peters et al. (52) performed a randomized intervention in children with dyslexia. The authors' objective was to evaluate if five hours of training with the Fruit Ninja^®^ game improved some reading skills in children. The short duration of the training, only five hours, did not lead to significant improvements in fixation patterns. Participants were young, around 10 years old, and compared between a group playing Fruit Ninja^®^ with and without using an eye tracker as a device in the experimental group.

Jeong et al. (34) had a group of experts and a group of non-experts playing the video game StarCraft^®^ to measure subjects’ ability to switch tasks using eye movements. The higher percentage of fixations in non-expert players indicates that they required longer fixations, the authors also concluded that the duration of fixations in AVGP was shorter than in NVGP.

Li et al. (39) used a visual search task in which participants had to find (all of) the letter ”T’s” (in a random array), using the letter “L” as distracting elements. Results showed that the duration of fixation in AVGP was significantly shorter than in NVGP, for all stimulus sizes used. Participants in AVG group had played more than 14 hours/week in action video games, and NVGP less than 2 hours/week. These results indicate that AVGP may have a time advantage over NVGP, since with a reduced fixation time they can obtain similar results in a shorter period of time.

#### Number

In the experiment by Li et al. (39), the number of eye fixations was significantly lower in AVGP than in NAVGP. Delmas et al. (17) found that distractors affected the number of eye fixations only in the AVGP, compared to NAVGP. Both groups performed a visual search task over a League of Legends^®^ wallpaper. The experiment of Schenk et al. (56) was an adapted version of the visual categorization task of Cook & Smith (16). Colored rings were used as stimuli and participants had to indicate whether they belonged in one of two categories depending on the combination of colors. As expected and due to the type of stimulus, one of the two segments, which was decisive for the correct categorization, presented a higher rate of fixations at the end of the experiment. This finding indicates a learning process that occurs in both groups (AVGP and NVGP), as the participants learn that the entire stimulus should not be explored. Compared to non-gamers, AVGP exhibited more central fixations, possibly indicating covert peripheral processing.

#### Distribution

Azizi et al. (5) investigated the influence of AVG on the behavior of eye movements during visual search, differentiating between more natural scenes compared to scenes similar to those appearing in video games. They trained a group of non-gamers for ten hours with Call of Duty (COD): Modern Warfare II^®^ and another group of non-gamers for ten hours with a card game. They found a reduced vertical distribution of fixations in the AVG-trained group, but only in the game's people-counting task (which consisted of counting the number of people that were in the picture) and not in natural picture search. The group trained with the AVG were learning where to look for targets in the game, but there was no evidence that this transferred to more natural domains.

#### Saccadic eye movements

The characteristics of saccadic movements are the most studied and compared between these two population groups since they are closely related to visual attention (37).

#### Gain

In a study by Chisholm & Kingstone (12), a difference in saccade gain appeared when a distractor element was introduced. AVGP made fewer saccadic movements towards the distractor elements and therefore their gain was greater. Another study from the same authors showed that AVGP made fewer incorrect saccades towards the distractor element (37.7%) than NVGP (47.5%). In the study by Heimler et al.(31), participants performed a visual search task in which they were asked to make a saccadic movement, as fast as possible, towards the target. They found that AVGP were less accurate compared to NVGP. These results are in line suggesting differences in the strategies adopted to solve the tasks in AVGP versus NVGP.

Diarra et al. (20) also investigated the gain of saccades. In their randomized intervention study in older adults (mean age around 60 years old), one group was trained on the game Super Mario 64^®^ for 6 months, and compared between a group learning piano lessons and a control group. AVG improved the gain of antisaccades during a specific designed task, which are eye movements in the opposite direction to the presented target, whereas the participants that were not trained on the game showed no improvement in saccade gain. Furthermore, improved gain was observed after three months of training, and performance remained stable after six months of post-training.

#### Latency

Chisholm et al. (14) and Chisholm & Kingstone (13) found no differences in saccadic latency comparing AVGP and NVGP. Diarra et al. (20) compared the reaction time of both antisaccades and prosaccades between a group of people who trained with video games for six months, an active control group who learned to play the piano for six months, and a passive control group. No significant differences in saccade latency were found between the three groups.

Schenk et al. (56) studied the mean first saccadic latency and found no differences between AVGP and NVGP on prosaccades. An increase in saccadic latencies was observed during the course of the experiment, which could indicate a learning process regarding stimulus characteristics in both groups. In contrast, Heimler et al. (31) found that AVGP were faster in executing saccadic eye movements compared to NVGP. Participants were asked to make the saccadic movement as fast as possible. Koposov et al.(36) used the popular multiplayer video game Counter-Strike: Global Offensive^®^ to measure saccadic latency with an eye tracker while playing the videogame. Mean and median saccadic latency values were smaller for professional players than for novice players for all types of targets.

#### Number

Schenk et al. (56) detected a decrease in the number of saccades from the beginning to the end of the experiment in all participants, without significant differences between AVGP and NAVGP, in a specific designed task. Jeong et al. (34) demonstrated that the saccadic ratio (a calculated metric from the authors, which was the number saccades and fixation events in a specific region and time) was higher and the fixation time shorter in the group of expert AVG players. The authors' interpretation of these results is that experts were able to change eye position faster and accumulate more information with shorter fixations. The result of fixation areas indicates that expert players placed more importance on overall flow than less skilled players.

#### Speed

Li et al. (39) found no significant differences between AVGP and NVGP in saccade speed during a visual search task that was carried out in the first part of the experiment. In the second part of the experiment, where central vision was differentiated from peripheral vision, AVGP had a significantly faster saccade speed in central vision, but no differences were found in peripheral vision. Jeong et al.(34) found faster saccades in expert players compared to non-expert players, while playing Starcraft^®^, without significant differences in the saccadic amplitude or length. In Jeong’s experiment, players were asked to change tasks and carry out simultaneous actions that required a high level of ability.

#### Amplitude

Li et al. (39), Azizi et al. (5), and Jeong et al. (34) studied the amplitude of saccadic eye movements. None of them found significant differences between the two groups. Li et al.(39) found that AVGP showed a greater amplitude and speed than NVGP, although these differences between groups were not statistically significant. The authors speculated that this advantage may be due to wider peripheral vision or a greater ability to process information. Azizi et al. (5), in the second part of the experiment, found no significant difference in the amplitude of saccades between experienced AVGP and NVGP before or after training in either task.

#### The curvature of the spatial trajectory

West et al. (63) evaluated a group of AVGP and another group of NVGP to find differences in oculomotor control. In their study, the measurement of choice was the curvature of the spatial trajectory of saccades since this parameter is independent of reaction time. Participants had to make a saccadic movement towards the target stimulus while being able to ignore a distractor. The trajectory of deviations toward or away from the distractor was measured to see if there were differences between the two groups. Saccadic curvature was calculated using the quadratic method proposed by Ludwig & Gilchrist (43). Although the effect of experience on gain was limited to the second half of the study, differences in saccadic trajectories between AVGP and NVGP were associated with better overall task performance by AVGP.

#### Pursuit eye movements

The only study found that evaluated pursuits is the work done Yee et al. (65). Three different groups; professional athletes, AVGP and a control group were compared in dynamic visual acuity and in pursuit eye movements performance with eye-tracker technology. In their experiment they used a range of velocities of 5, 10, 20 and 30 º/s. AVGP were defined as at least 1 hour for at least 6 months playing video games such as Counter Strike^®^, Unreal Tournament^®^ or Call of Duty^®^. No significant differences were found in eye pursuits between the three groups in terms of speed and precision.

#### Study of bias

The studies by Peters et al.(52), Diarra et al.(20) and Azizi et al.(5) were evaluated with the ROB-2 tool, in accordance with the recommendations of the Cochrane guide (32). The results of the risk of bias were divided into five domains, 1: bias arising from the randomization process, 2: bias due to deviations from intended intervention, 3: bias due to missing outcome data, 4: bias measurement of the outcome, and 5: bias in selection of the reported result. Overall, the studies of Peters et al.(52), and Diarra et al.(20) resulted in a low risk of bias, whereas the study of Azizi et al.(5), failed domain 2.

To summarize the results for all the studies included in Table 1, the additional Table 2 includes the main findings analyzed.

**Table 2.** Summary of the main findings from the studies analyzed. AVGP: Action Video Game Players. NVGP= Non-Video Game Players. A more detailed description about each study in terms of design, methodology and videogames used, can be found at Table 1.

	**AVGP = NVGP**	**AVGP > NVGP**	**AVGP < NVGP**
	**Fixations**		
	Duration		
Chisholm & Kingstone.(2012)	**x**		
Azizi et al.(2017)	**x**		
Peters et al.(2019)	**x**		
Jeong et al.(2022)			**x**
Li et al.(2022)			**x**
	Number		
Li et al.(2022)			**x**
Peters et al.(2019)	**x**		
Delmas et al.(2022)		**x**	
Schenk et al.(2020)		**x**	
	Distribution		
Azizi et al.(2017)			**x**
	**Saccades**		
	Gain		
Chisholm & Kingstone (2012)		**x**	
Chisholm & Kingstone (2015)		**x**	
Heimler et al.(2014)			**x**
Diarra et al.(2019)		**x**	
	Latency		
Chisholm & Kingstone (2012)	**x**		
Chisholm & Kingstone (2015)	**x**		
Diarra et al.(2019)	**x**		
Schenk et al.(2020)	**x**		
Heimler et al.(2014)			**x**
Koposov et al. (2020)			**x**
	Number		
Schenk et al.(2020)	**x**		
Jeong et al.(2022)		**x**	
	Speed		
Li et al.(2022)		**x** (only in central vision)	
Jeong et al.(2022)		**x**	
	Amplitude		
Li et al.(2022)	**x**		
Azizi et al.(2017)	**x**		
Jeong et al.(2022)	**x**		
	Curvature		
West et al. (2013)	**x**		
	**Pursuits**		
	Speed and Precision		
Yee et al. (2021)	**x**		

## Discussion

The goal of this systematic review was to investigate the possible modifications seen in eye movements through either regularly playing or treatment with AVG. Of the thirteen studies (N=13) selected, ten (n=10) were cross-sectional and three (n=3) were interventional. Two of the authors (A.M.V and M.A) conducted the study selection and data collection.

Eye movement behavior and patterns are intricately linked to cognitive processes, particularly visual attention (35). Existing literature shows a connection between playing AVG and the modification of eye movement behaviors (5; 12; 31; 44) and consistently shown that individuals engaged in AVG, referred to as AVGP, demonstrate heightened visual attention abilities (2; 3; 7; 6; 27, 28). This hypothesis finds support in the broader context of AVG benefits, as previous research has highlighted the capacity of AVG training to improve reading performance in individuals with dyslexia (21; 23; 52). Therefore, by drawing on the known associations between eye movements, cognitive processes, and the specific impacts of AVG, our study explored the potential link between AVG playing and modifications in eye movement behavior.

### Differences in eye movements between high-level experienced AVGP compared with NVGP

The results of the cross-sectional studies included in the present systematic review showed that AVGP might have different patterns in eye fixations during the videogame, exhibiting shorter duration of fixations (34; 39). Theoretically, a reduced fixation time indicates an advantage in a game; however, this result depends greatly on the type of task and varies from one study to another; hence, we cannot assume that playing a videogame decreases the duration of eye fixations. In general, the number of fixations can be influenced by the complexity of the stimulus and the visual task requirements, and can be influenced by individual factors such as attention, cognitive load, and visual expertise. This effect is in line with some studies that show that less number of fixations are related with more expertise in the task that they see (4; 10; 45), possibly related with a higher visual span in experts during the task (55). Interestingly, an increased visual attention span has been observed in AVGP (2). Besides, it is not clear yet that AVGP exhibits a shorter duration of fixations during the videogame. Both duration and number are dynamic, and depend on various factors including the nature of the visual stimuli and the task being performed (48). Then, it seems that these possible differences observed are from the experience and learning itself, but again only one study supports this. In terms of saccades, less saccade amplitude is related to more difficult tasks (66), more velocity with more task difficulty (24), and more distraction and expertise during the task with a decrease of latency (58). Thus, it seems plausible that experts in AVG can exhibit differences in saccade metrics during the video games, but this does not transfer to naturalistic tasks.

A possible explanation comes from differences in methodology between studies. As Stewart et al.(61) stated: “given the high costs and difficulty in running full intervention studies, cross-sectional designs are often used by researchers to determine whether a full-scale intervention is warranted”. Further, more randomized intervention studies are needed to draw firm conclusions in this area.

High variability in the inclusion criteria across studies was found in AVG and NVGP groups between cross-sectional studies. The critical outcome measures in these studies are theorized to show a difference in performance between these two self-selected groups (i.e., whether AVGP shows better performance than NVGP. The most commonly used criterion for including a participant in the AVGP group or expert, is the number of hours per week they spend playing this type of video game. Some authors consider the number of hours played in the last six months, others in the last year, and some in the past two years. The threshold number of hours per week is also variable and ranges from a minimum of three hours per week to more than twenty hours per week. Other studies use as a criterion the rankings in the different leagues that exist.

For instance, Delmas et al.(17) included in the AVGP group a ranking position in the League of Legends^®^ video game, in contrast to participants that never played in League of Legends^®^, which were included in the NVGP. Koposov et al., 2020 compared amateur players with professional player experts in the videogame Counter Strike^®^. Different criteria among experts and non-experts in the same videogame can also affect the eye movement patterns, which are also highly dependent on the task used (see Table 1 for more information).

Regarding inclusion in the NVGP group, researchers usually select individuals who have played little or no video games in the last six months to two years. The type of recruitment done in these cross-sectional studies is an open one in which AVG participants know that they are included in the study because they play this type of video game and, in addition, participants themselves declare the experience they have in games. Therefore, there is a strong self-selection bias in these types of research. Another important factor is the gender bias in some studies (for more information see Table 1). For instance, in Schenck et al.,^56^ study, the NVGP group had more females (14) than males (2) compared with the AVG group, which was 14 males and 2 females. Yee et al. (65) used in NAVGP 13 females and 7 males, in contrast with 2 females and 9 males in the AVGP. However, in general, most of the analyzed studies have a gender bias in AVGP and NVGP (31; 39; 65), which could be explained by the fact that males generally play more AVG than females (29), and may make gender homogeneity gender criteria difficult for AVGP and NVGP groups.

### Changes in eye movements before and after cognitive treatment using AVG

Only 3 intervention studies were identified in this systematic review. Our hypothesis was if training with AVG transfers to a change in eye movement behavior. From these 3 studies, only Diarra et al.(20) found an improvement in saccade gain and antisaccades while playing Super Mario 64^®^ for 6 months. Another issue to consider in these intervention studies is the dose-response effect, i.e, the number of hours of training with the AVG to have an impact on eye movements. These 3 studies used different training times with AVG, 10 hours with Call of Duty^®^ in Azizi et al.(5), 5 hours with Fruit Ninja^®^ in Peters et al.(52), and 6 months training with Super Mario 64^®^ (with no total amount of hours training detailed) in Diarra et al.(20). In their review, Chopin et al.(15) found that in the case of AVG and perception, intervention studies with more than twenty hours of training were needed (15).

### Eye movement metrics during the videogame or during motor tasks

An important issue comparing eye movement behavior and metrics in saccades, fixations, and pursuits in the studies compared in this study, is whether these differences were analyzed during the videogame or during specific motor tasks. Differences during the videogame reflect attentional strategies, and differences during specific motor tasks reflect the states of ocular motor programing. Among the studies, only Jeong et al. (34) and Koposov et al. (36) compared the differences in eye movement during the videogame, and West et al. (63), Chisholm & Kingstone (12), Chisholm & Kingstone (13), Li et al. (39), Schenk et al (56), Delmas et al (17), Heimler et al (31), Diarra et al (20), and Yee et al (65) used a designed motor task. Eye movement metrics analyzed in Peter et al. (52) were during reading performance, and Azizi et al. (5) used a specific oculomotor task of visual search, with different backgrounds, among them with the action video game. In most investigations included in this review, the study of eye movements was not the main goal but a secondary objective or a consequence of measuring other data. Therefore, it would be necessary to design research whose main objective was to study eye movements to obtain different results. Again, we cannot draw a proper conclusion about the possible transference in eye movement metrics and patterns with AVG training.

### Conclusions

Despite the amount of literature that shows an improvement in visual attention in both cross-sectional and training studies with AVG (7; 6; 8; 22; 28), in the domain of eye movements, no conclusion can be given that either regularly playing or training with AVG can change eye movement metrics in terms of fixations, saccades, and pursuits, either playing during the videogame or in naturalistic tasks. The results of the studies examined in this systematic review were highly dependent on the task used to measure eye movements. There is a lack of consensus among the different authors about which measures and characteristics of eye movements are of interest to evaluate the effects of AVG. Oculomotor metrics are highly dependent on the task and therefore, they need to be understood in the context of the task in hand. For example, visual search tasks or oculomotor capture will show different patterns in saccadic and fixation parameters. Since the studies included in the present review assess eye movements in different tasks it cannot be concluded that eye movements improve from either regularly playing or training with AVG.

Based on our systematic review, it seems probable that playing AVG can have a positive impact in those metrics that facilitate the attentional resources of gamers, which in part benefits performance on the videogame, but it can come from the experience itself that changes eye movement behavior during the task (45; 55). It is not clear yet the possible transference in eye movement behavior after training with AVG. More intervention studies with more consensus distinction in inclusion criteria between participants playing action or non-action video games, and task design consensus to evaluate eye movements are needed to draw firm conclusions. Hence, our analysis shows different results from different authors in the distinct metrics of eye movement behavior, either comparing AVGP and NVGP, or training with and without AVG. For instance, two studies showed that AVGP has less duration in fixations, although 3 studies did not observe any differences (see Table 2). From our analysis, we can draw some conclusions about these discrepancies. First, more interventional studies are needed to obtain more reliable results in eye movement analysis. Second, it is important to use the same task with specific aims to study eye movement behavior and metrics such as fixations, saccades or pursuits in future studies. Finally, more consensus between either the action and non-action video games, and AVGP and NVGP is needed.

In conclusion, despite the scientific evidence showing that playing AVG can enhance visual attention, reading performance and visual search, the present systematic review concludes that playing regularly or training with action video games is not likely to produce changes in eye movements metrics, based on the literature research analyzed.

### Ethics and Conflict of Interest

The authors declares that the contents of the article are in agreement with the ethics described in http://biblio.unibe.ch/portale/elibrary/BOP/jemr/ethics.html and that there is no conflict of interest regarding the publication of this paper.

### Acknowledgements

This publication is part of the project PID2020-112527RB-I00, funded by MCIN/AEI/10.13039/501100011033.

## supplementary material


